# Identification of Heat-Responsive Genes in Guar [*Cyamopsis tetragonoloba* (L.) Taub]

**DOI:** 10.1155/2020/3126592

**Published:** 2020-06-19

**Authors:** Aref Alshameri, Fahad Al-Qurainy, Abdel-Rhman Gaafar, Salim Khan, Mohammad Nadeem, Saleh Alansi

**Affiliations:** Department of Botany and Microbiology, College of Science, King Saud University, Riyadh 11451, Saudi Arabia

## Abstract

The threat of heat stress on crop production increased dramatically due to global warming leading to the rise on the demand of heat-tolerant crops and understanding their tolerance. The leguminous forage crop Guar [*Cyamopsis tetragonoloba* (L.) Taub] is a high-temperature tolerant plant with numerous works on its tolerance at morph-physiological levels but lack on molecular thermotolerance level. In the current study, the differential gene expression and the underlying metabolic pathways induced by heat treatment were investigated. An RNA-Seq study on Guar leaves was carried out to estimate gene abundance and identify genes involved in heat tolerance to better understand the response mechanisms to heat stress. The results uncovered 1551 up- and 1466 downregulated genes, from which 200 and 72 genes with unknown function could be considered as new genes specific to guar. The upregulated unigenes were associated with 158 enzymes and 102 KEGG pathways. Blast2GO, InterProScan, and Kyoto Encyclopaedia of Genes and Genomes packages were utilized to search the functional annotation, protein analysis, enzymes, and metabolic pathways and revealed hormone signal transduction were enriched during heat stress tolerance. A total of 301 protein families, 551 domains, 15 repeats, and 3 sites were upregulated and matched to those unigenes. A batch of heat-regulated transcription factor transcripts were identified using the PlantTFDB database, which may play roles in heat response in Guar. Interestingly, several heat shock protein families were expressed in response to exposure to stressful conditions for instance small HSP20, heat shock transcription factor family, heat shock protein Hsp90 family, and heat shock protein 70 family. Our results revealed the expressional changes associated with heat tolerance and identified potential key genes in the regulation of this process. These results will provide a good start to dissect the molecular behaviour of plants induced by heat stress and could identify the key genes in stress response for marker-assisted selection in Guar and reveal their roles in stress adaptation in plants.

## 1. Introduction

The extreme high temperatures, and other changes in the global climate, have resulted in destructive damage to crop production [1, 2]. The unending rise of the average climate temperature by 6.9*°*C at the end of this century due to global warming is expected to reduce crop productivity by 15-35% based on the increment of 3-4*°*C [3, 4].

Heat stress damages plant cellular components through different mechanisms as a consequence of temperatures that far exceed ideal growth conditions [5]. Heat stress commonly impairs photosynthetic efficiency, and reduced temperature-induced water content has negative effects on cell division and growth [6]. Plants have evolved complex and diverse systems as sessile organisms to cope with this stress [7]. The mechanisms of heat tolerance have been gradually revealed over the past few years. The tolerance is determined by effective coordination between various physiological processes, metabolic pathways, and gene networks under heat stress. Identifying these underlying mechanisms will promote our understanding on how the plant responds to climate change and aid in the development of more adapted crops to stress conditions. Moreover, Novel processes or genes may exist in different heat-tolerant species involve in heat response. These genes enhance the tolerance of plants to heat and provide more choices for the improvement of heat tolerance in crops. However, considering the variety of these species, our understanding of the mechanism underlying heat tolerance is limited [7].

The breakthroughs in next-generation sequencing, especially for Illumina RNA-Seq, is rapidly becoming the method of choice for transcriptional profiling experiments and offered new opportunities for comprehensive transcriptomic analyses in nonmodel plants. RNA-seq has been widely used to research the responses of plants to abiotic stress [8–10] and has been applied to several heat-tolerant plants to uncover their mechanisms of heat tolerance [10–12]. This technology can reveal candidate genes and key pathways involved in heat tolerance by analyzing differentially expressed genes (DEGs) and functional annotation. Transcriptome analysis reveals that several levels of biochemical and molecular responses are associated with heat tolerance in Poaceous species [10–15]. Differential expression under heat stress also investigated in Solanaceous species. [16–24]. In leguminous plants, there are few studies regarding the DEGs induced by heat stress [25, 26]. About 5 percent of the transcriptome of the plant is upregulated under heat stress in plants and, although often heat stress-induced, chaperones are only a small part of the overall heat shock response [27, 28]. Most transcripts are genes that function in translation, metabolism, transcription, control, and environmental stress response, as well as in processes such as calcium, phytohormone, sugar and lipid signals, or protein phosphorylation [29].

Guar [*Cyamopsis tetragonoloba* (L.) Taub] is a leguminous forage and industrial crop which is known for its high tolerance to heat stress. While numerous works have been conducted to measure its tolerance at morphological and physiological levels; however, systematic investigation into its molecular thermotolerance and the gene regulation network under stress conditions, using whole-genome gene expression profiling methods, is indispensable.

The molecular mechanisms involved in plant heat stress response need to be determined in order to understand how plants react and adapt to heat stress and to produce crops with improved thermotolerance ability to survive and thrive to grow under heat stress conditions. In the current study, gene expression profiling was conducted in Guar leaves in response to heat treatment (42°C) at preflowering stage for leaves of heat-tolerant guar accession “PWP 5595” compared with the control (25°C) using RNA-seq to investigate metabolic adjustment and identify the genes that may play a vital role in heat adaptation in Guar. The present work provides important data for understanding the heat tolerance mechanism of this crop plant and establishes an important transcriptomic database for further study.

## 2. Materials and Methods

### 2.1. Plant Material

Seeds of *C. tetragonoloba* accession BWP 5595 (highly heat stress) were sown in pots containing a mix of 1 : 1 : 1 peat moss, perlite, and soil and allowed to grow regularly for 35 days. Heat stress treatment (GH) at 42°C and control (GC) at 25°C with three biological replicates were applied to plants for three weeks later. Completely randomized design (CRD) was utilized to arrange all treatments with three biological replicates for each. Leaf samples were frozen directly with liquid nitrogen and stored at -80°C for subsequent RNA-Seq.

### 2.2. RNA Extraction, Library Construction, and Sequencing

Total RNA from leaf samples was extracted following the manufacturer's guidelines of the RNeasy® Plant Mini Kit (QIAGEN). DNase I, Bovine Pancreas (Biomatik) was utilized to remove DNA contamination. Agilent 2100 Bioanalyzer was used to assess the RNA integrity number (RIN). RNA samples with recommended purity and integrity were shipped to the Macrogen Inc. (http://www.macrogen.com). cDNA library was constructed using Illumina TruSeq® Stranded mRNA kit. Paired-end sequencing was carried out using Illumina HiSeq 2500 platform.

### 2.3. Read Quality Control and Adapter Removal

The quality of the raw sequence data was checked using FASTQC V 0.11.5 [30]. The adapter sequences were filtered, and low-quality reads were trimmed by Trimmomatic V 0.36 [31]. Random sequencing errors were corrected using Rcorrector V 1.0.3 [32].

### 2.4. Gene Quantification

In our previous work [33], a comprehensive de novo guar transcriptome assembly was utilized as a reference based on the longest isoforms of the transcriptome 61508 transcripts. To estimate gene abundance, an alignment-based approach (aligning reads to the reference transcript assembly) was carried out via RSEM V1.1.13 (RNA-Seq by Expectation-Maximization; [34]). A matrix of counts and a matrix of normalized expression values were constructed using the gene-level abundance estimates for each of sample.

### 2.5. Quality Check of Conditions and Biological Replicates

The correlation and relationships among the stress conditions were investigated using the script “PtR” (Perl-to-R) by comparing and generating a correlation matrix for each condition replicates. Principal Component Analysis (PCA) (http://github.com/trinityrnaseq/trinityrnaseq/wiki/) was carried out to visualize and explore relationships among the sample replicates.

### 2.6. Differential Expression Analysis

Differentially expressed genes were identified and clustered according to expression profiles through EdgeR V3.08 (Empirical Analysis of Digital Gene Expression Data in R) Bioconductor package [35] under TMM normalization (Trimmed Mean of M values; [36]). R package [30] and R studio [31] are used to generate MA and volcano plots. Expressed genes were only considered as significantly regulated DE with parameters set at the cut off value of FDR (False Discovery Rate) [32] with high stringency *p* value 0.001, and absolute FC (fold-changes) [37] ≥2. Heatmaps of heat stress correlation matrix were analyzed by R package and R studio. The DE genes shown in the heatmap above were partitioned into gene clusters with similar patterns of expression at Ptree of 70, *p* value of 0.001, and FC of 2.

### 2.7. Gene Ontology (GO) Enrichment Analysis and Metabolic Signalling Pathway Analysis

The professional version of Blast2GO software suite v4.1 (https://www.blast2go.com/; [38–40]) was utilized to carry out homology searches (BLASTX and BLASTN) of unique sequences and functional annotation by gene ontology enrichment analysis (GO; http://www.geneontology.org), protein sequence analysis & classification (InterPro, EBI, https://www.ebi.ac.uk/interpro/), enzyme classification codes (EC), and Kyoto Encyclopedia of Genes and Genomes (KEGG, http://www.genome.jp/kegg/). Sequences were blasted against a nonredundant (nr) protein database belongs to the National Center for Biotechnology Information (NCBI, https://www.ncbi.nlm.nih.gov/) via BLASTx-fast using the default parameters. Interpro was scanned in parallel with the blasting step and gene ontology mapping and annotation. BLAST2GO has also been used for assigning the genes biological functions, cellular components, cellular processes, and other useful statistics.

### 2.8. Identification of Transcription Factors (TFs)

The TF database PlantTFDB v4.0 (http://planttfdb.cbi.pku.edu.cn/download.php; [41]) was used as a reference TF database for the identification of transcription factor families related to heat stress. Putative TFs in *C. tetragonoloba* were identified using BLASTx with a cut-off *E*-value of 1 × 10^−5^ (Chen & Li, 2017) and best hit in *Arabidopsis thaliana*.

## 3. Results

### 3.1. Sequencing and Quality Control

High-quality RNAs of heat stress and control conditions were sequenced. In total, 110.7 million paired-end raw reads (~22 Gbp) with an average read length of 100 bp were generated from the targeted samples (Table 1). The GC content ranged between 43.98% (H3) and 44.77% (C1). The ratio of reads that have a Phred quality score of over Q30 ranged from 94.58% (C1) to 95.3% (H3) indicating high-quality reads. After checking the quality of reads, the adapter sequences were removed. The low-quality reads with or without ambiguous sequences “N” were trimmed which resulted in the dropping of about 2.63% paired reads. The remaining reads were processed for correction of random sequencing errors. Quality control reflected extremely high-quality reads after the trimming and bases correction procedures.

### 3.2. Quality Check for Heat Stress Condition and Biological Replicates

Quality control of biological replicates showed high homogenization between replicates either in the control or in the heat stress as shown in the Supporting Figures 1–8. The biological replicates are more highly correlated within than among treatments (Supporting Figure 9 and Supporting Table 1). Principal component analysis showed clustering of the biological replicates closely according to treatment type (Supporting Figure 10 and Supporting Table 2).

### 3.3. Identifying Differentially Expressed Genes

The output from running the DE analysis resulted in identifying of logFC, logCPM, *p* value, and FDR for each gene. Furthermore, MA and volcano plots of DEGs are shown in Supporting Figure 11. The genes that are expressed most differently (FDR of 0.001 and fold-changes of 2) were extracted and clustered across the heat stress and control according to their differential expression patterns. The expression matrix subset for genes upregulated in the heat stress and the control are calculated. All features found DE in any of the pairwise comparisons consolidated into a single expression matrix. A Pearson correlation matrix for pairwise sample comparisons based on the set of DE genes is shown in Supporting Table 3. The clustered heatmap revealed the above sample correlation matrix at *p* value of 0.001 and FC of 2 is illustrated in Supporting Figure 12. The DEGs of heat stress (GH) via control (GC) clustered heatmap at *p* value of 0.001 and FC of 2 are shown in Supporting Figure 13. Cutting the hierarchically clustered gene tree at 60% height of the tree resulted into partitioning genes into two clusters with similar expression patterns which represent 1551 upregulated DEGs and 1466 downregulated DEGs responsive to heat stress (Supporting Figure 14). The sequences of these genes were extracted by our own script that prepared for this purpose depending on the IDs of the genes. These two gene clusters were further analyzed, as shown below.

### 3.4. Analysis of Upregulated Genes Responsive to Heat Stress

#### 3.4.1. Blasting, Mapping, and Annotation

The total of 1551 upregulated genes was subjected to analysis using BLAST2GO. Examples of upregulated DE heat-responsive genes in guar are listed in Table 2 and Supplementary excel file (available here). Out of those genes, 1550 (99.87%) were with InterProScan, 1351 (87.11%) blasted, 1084 (69.85%) mapped, and 1030 (66.41%) annotated. The extra unblasted 200 genes could be considered as new genes exclusive to guar that could be upregulated significantly under heat stress. These new genes might be a useful material for future researches. *E*-value distribution shows that all of the 26599 hits were at *E* value ≥ 1*e*-4 and the most significant hits (28%) were at *E* value ≥ 1*e*-180 indicating high hit rate and very low random background noise.

A considerable amount of mapping data (88.37 percent of unigenes with mapping data) was extracted from the UniProt Knowledgebase (UniProtKB) database, followed by Arabidopsis Information Resource (TAIR; 2.78%), Protein Data Bank (PDB; 0.06%), and GR_protein (0.01%).

#### 3.4.2. Protein Sequence Analysis and Classification (InterProScan; IPS)

**Figure 1:**
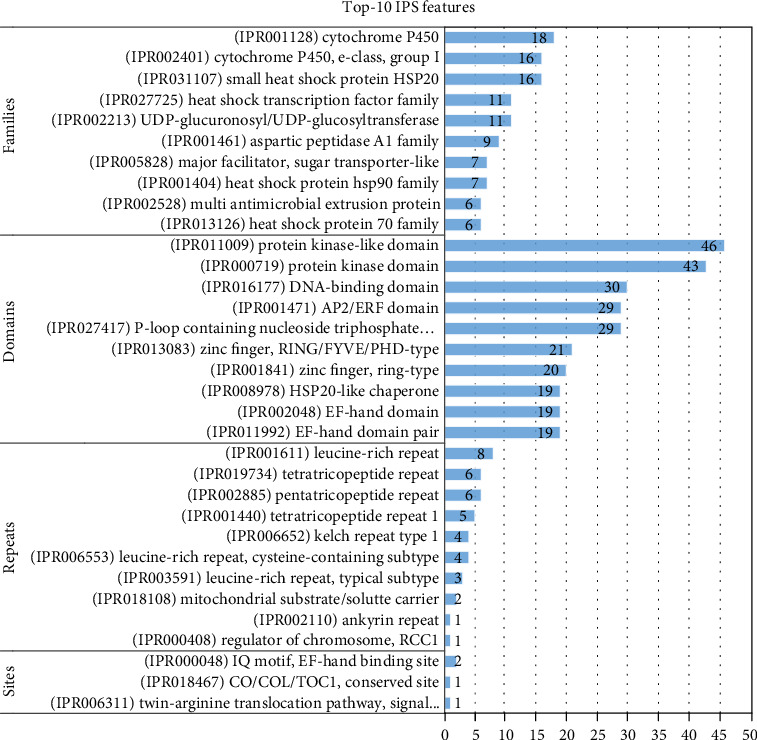
Top 10 IPS families, domains, repeats, and sites for upregulated DEGs responsive for heat stress.

Out of 1551 upregulated DEGs, there were 1192 (76.80%) that had IPS and 668 (43.04%) of them had GOs. A total of 301 protein families were found (Figure 1). The family (IPR001128) Cytochrome P450 had the largest number of unigenes (18 unigenes) followed by (IPR031107) Small heat shock protein HSP20 (16 unigenes), (IPR002401) Cytochrome P450, E-class, group I (16 unigenes), (IPR002213) UDP-glucuronosyl/UDP-glucosyltransferase (11 unigenes), (IPR027725) Heat shock transcription factor family (11 unigenes), (IPR001461) Aspartic peptidase A1 family (9 unigenes) and (IPR001404) Heat shock protein Hsp90 family (7 unigenes), (IPR005828) Major facilitator, sugar transporter-like (7 unigenes), and (IPR013126) Heat shock protein 70 family (6 unigenes). The residual 78 IPS families were related to 2-5 unigenes, and 214 IPS families related to a single unigene.

A total of 551 protein domains were detected (Figure 1). (IPR011009) Protein kinase-like domain matched with the largest number of unigenes (46 unigenes), followed by (IPR000719) Protein kinase domain (43 unigenes), (IPR016177) DNA-binding domain (30 unigenes), (IPR027417) P-loop containing nucleoside triphosphate hydrolase (29 unigenes), and (IPR001471) AP2/ERF domain (29 unigenes). Other domains 234 domains matched with 2-21 unigenes and 311 domains matched with single unigenes. The following heat shock protein (HSP) domains were detected: (IPR020575) Heat shock protein Hsp90, N-terminal (7 unigenes), (IPR029047) Heat shock protein 70kD, peptide-binding domain (6 unigenes), (IPR000232) Heat shock factor (HSF)-type, DNA-binding (6 unigenes), (IPR029048) Heat shock protein 70kD, C-terminal domain (5 unigenes), (IPR001305) Heat shock protein DnaJ, cysteine-rich domain (2 unigenes), and (IPR006636) Heat shock chaperonin-binding (1 unigene).

A total of 15 IPS repeats were detected. (IPR001611) Leucine-rich repeat matched with the largest number of unigenes (8), followed by (IPR002885) Pentatricopeptide repeat (6 unigenes), and (IPR019734) Tetratricopeptide repeat (6 unigenes). Three detected IPS sites were (IPR000048) IQ motif, EF-hand binding site (2 unigenes), (IPR006311) Twin-arginine translocation pathway, signal sequence (1 unigene), and (IPR018467) CO/COL/TOC1, conserved site (1 unigene).

#### 3.4.3. Functional Annotation

**Figure 2:**
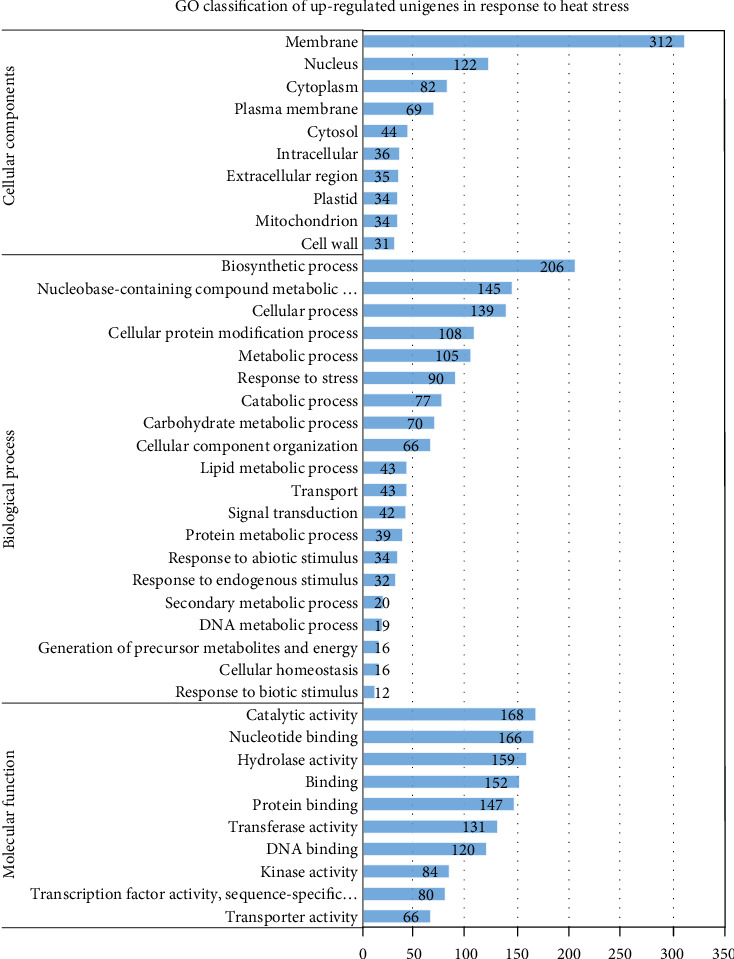
GO classification of the upregulated genes under heat stress.

Of the three-core GO annotation categories, biological processes (BP) comprised 37.88% of the total assigned annotations. Whereas molecular functions (MF) and cellular components (CC) comprised 37.96% and 24.16%, respectively. The GO terms with the largest number of assigned unigenes in the biological process (BP) category were biosynthetic process (206; 14.30%), nucleobase-containing compound metabolic process (145; 10.06%), cellular process (139; 9.65%), cellular protein modification process (108; 7.49%), metabolic process (105; 7.29), and response to stress (90; 6.25%) (Figure 2). Meanwhile, within the cellular component category (CC), the terms with the most unigenes were membrane (312; 33.95%), nucleus (122; 13.28%), cytoplasm (82; 8.92%), plasma membrane (69; 7.59%), and cytosol (44; 4.79). In the molecular function (MF) category, the terms with the most unigenes were catalytic activity (168; 11.63%), nucleotide binding (166; 11.50%), hydrolase activity (159; 11.01%), binding (152; 10.53%), protein binding (147; 10.18%), and transferase activity (131; 9.07%).

#### 3.4.4. KEGG Pathways Mapping

**Figure 3:**
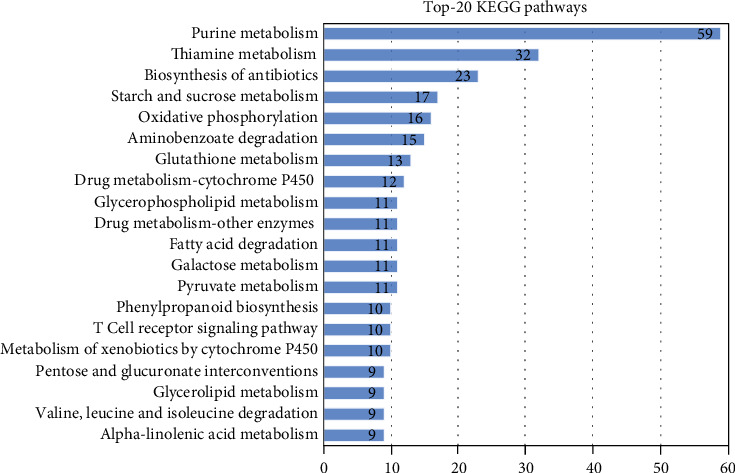
The top 20 KEGG pathways assignments in upregulated heat stress-responsive unigenes. The number of unigenes predicted to belong to each category is shown.

The KEGG pathways-based analysis indicated that 225 (14.49%) unigenes of the 1551 upregulated unigenes under heat stress obtained hits in the KEGG database, and those unigenes were associated with 158 enzymes and 102 KEGG pathways (Supporting Table 4). The top pathways are presented in Figure 3. Unigenes associated with Purine metabolism (59 unigenes) were most representative, followed by thiamine metabolism (32 unigenes) and biosynthesis of antibiotics (23 unigenes). An example of those metabolic pathways' maps is presented here (Supporting Figure 15).

**Figure 4:**
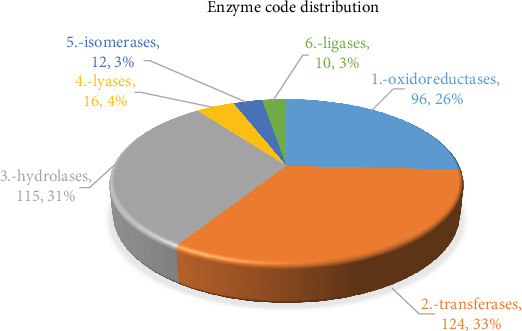
Enzyme code distribution of heat stress-responsive upregulated.

The 158 enzymes were further categorized into 6 main classes. As illustrated in Figure 4, Transferase enzymes represented the largest number of unigenes (124; 33%), followed by Hydrolases (115; 31%), Oxidoreductases (96; 26%), Lyases (16; 4%), Isomerases (12; 3%), and Ligases which represented the lowest number of unigenes (10; 3%). These 6 classes were categorized again to subclasses.

#### 3.4.5. Upregulated Transcription Factors (TFs)

**Figure 5:**
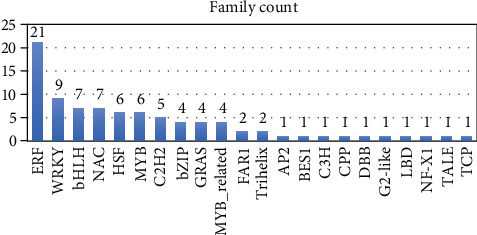
Distribution of upregulated transcription factors (TFs) under heat stress.

Transcription factors are important regulators that participate in the response to biotic and abiotic stresses. To better understand the molecular mechanism which regulates the heat stress response in *C. tetragonoloba*, 87 upregulated TFs were identified from DEGs according to the rules of family assignment illustrated in PlantTFDB. These upregulated TFs belong to 22 TF families. ERF family represented the most of TFS (21), followed by WRKY (9), bHLH (7), NAC (7), HSF (6), MYB (6), C2H2 (6), then the other families belonged 4 TFS or less (Figure 5).

The results and description of the best hit of these TFs against *Arabidopsis thaliana* are presented in Supporting Table 5. Five TFs in the heat shock factor (HSF) family were identified including heat shock factor 4 (HSF4), heat shock transcription factor A6B (HSFA6B), heat shock transcription factor B3 (HSFB3), heat shock transcription factor A4A (HSFA4A), and heat shock transcription factor B2A or heat shock factor 6 (HSFB2A).

### 3.5. Analysis of Downregulated Genes Responsive to Heat Stress

#### 3.5.1. Blasting, Mapping, and Annotation

The total of 1467 downregulated unigenes were subjected to analysis using BLAST2GO. Out of those unigenes, 1467 (100%) were with InterProScan, 1395 (95%) blasted, 1172 (84%) mapped, and 1135 (77.37%) annotated. The extra unblasted 72 unigenes could be considered as new genes exclusive to guar that could be downregulated significantly in response to heat stress. These new genes might be a useful material for future researches. *E*-value distribution shows that all of the 27445 hits were at *E* value ≥ 1*e*-4 and the most significant hits (41%) were at *E* value ≥ 1*e*-180 indicating a high quality of hits and very low random background noise. A considerable amount of mapping data (97.16% of unigenes with mapping information) was extracted from the UniProtKB database, followed by Arabidopsis Information Resource (TAIR; 2.78%), Protein Data Bank (PDB; 0.06%), and GR_protein (0.01%).

#### 3.5.2. Protein Sequence Analysis and Classification (IPS)

**Figure 6:**
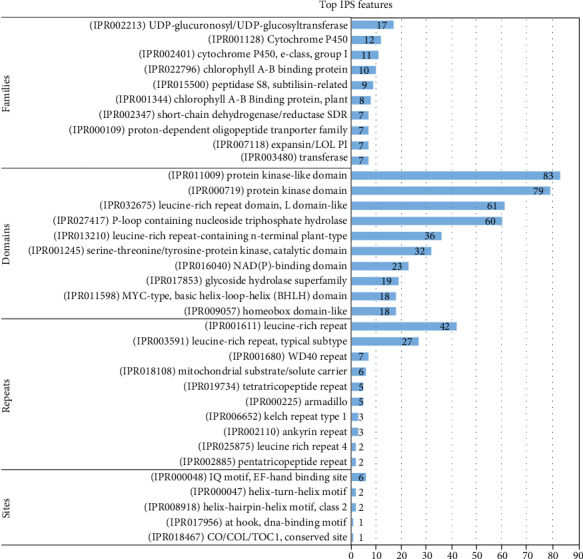
Top 10 IPS families, domains, repeats, and sites for heat stress-responsive downregulated unigenes.

Out of 1467 unigenes, there were 1298 (88.48%) that had IPS and 815 (55.56%) of them had GOs. A total of 353 IPS families were found (Figure 6). The family (IPR002213) UDP-glucuronosyl/UDP-glucosyltransferase had the largest number of unigenes (17 unigenes) followed by (IPR001128) Cytochrome P450 (12 unigenes), (IPR002401) Cytochrome P450, E-class, group I (11 unigenes), and (IPR022796) Chlorophyll A-B binding protein (10 unigenes). Furthermore, other families were related to 2-9 unigenes, and 250 IPS families related to a single unigene. A total of 647 domains were detected (Figure 6). (IPR011009) Protein kinase-like domain matched with the largest number of unigenes (83 unigenes), followed by (IPR000719) Protein kinase domain (79 unigenes), (IPR032675) Leucine-rich repeat domain, L domain-like (61 unigenes), (IPR027417) P-loop containing nucleoside triphosphate hydrolase (60 unigenes), (IPR013210) Leucine-rich repeat-containing N-terminal, plant-type (36 unigenes), and (IPR001245) Serine-threonine/tyrosine-protein kinase, catalytic domain (30 unigenes). Other domains 309 domains matched with 2-23 unigenes and 332 (51.3%) domains matched with single unigenes.

A total of 109 IPS repeats were detected. (IPR001611) Leucine-rich repeat matched with the largest number of unigenes (42; 38.53%), followed by (IPR003591) Leucine-rich repeat, typical subtype (27; 24.77%). Five IPS sites were detected: (IPR000048) IQ motif, EF-hand binding site (6 unigenes), (IPR000047) Helix-turn-helix motif (2 unigenes), (IPR008918) Helix-hairpin-helix motif, class 2 (2 unigenes), (IPR017956) AT hook, DNA-binding motif (1 unigene), and (IPR018467) CO/COL/TOC1, conserved site (1 unigene).

#### 3.5.3. Functional Annotation

**Figure 7:**
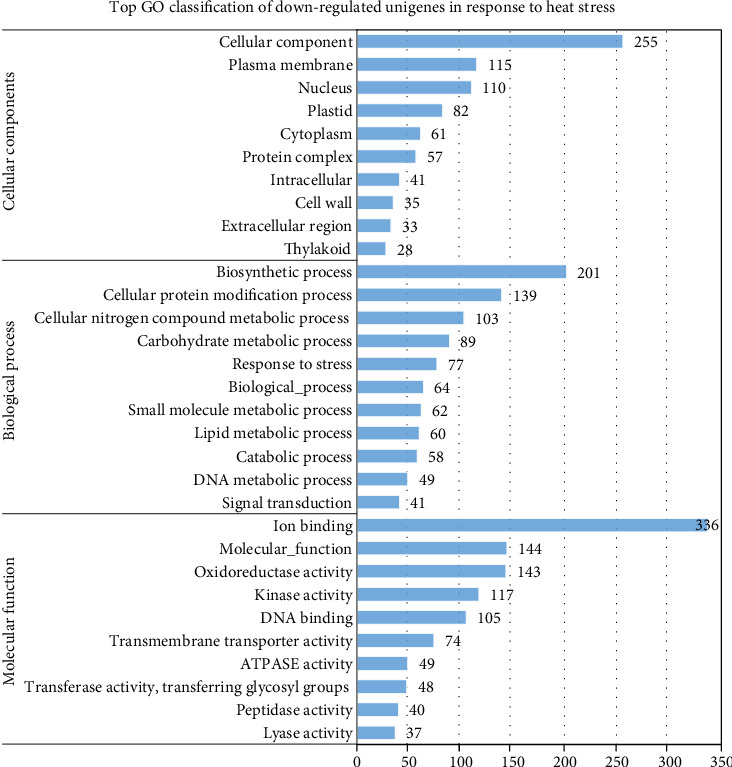
GO classification of the downregulated genes detected in leaf tissues of the guar accession under heat stress.

Of the three-core GO annotation categories, biological processes (BP) comprised 36.94% of the total assigned annotations, whereas molecular functions (MF) and cellular components (CC) comprised 36.79% and 26.27%, respectively. The GO terms with the largest number of assigned unigenes in the biological process (BP) category were biosynthetic process (201; 13.99%), cellular protein modification process (139; 9.67%), cellular nitrogen compound metabolic process (103; 7.17%), carbohydrate metabolic process (89; 6.19%), and response to stress (77; 5.36%) (Figure 7). Meanwhile, within the cellular component category (CC), the terms with the most unigenes were cellular component (255; 24.95%), plasma membrane (115; 11.25%), nucleus (110; 10.76%), plastid (82; 8.02%), and cytoplasm (61; 5.97). In the molecular function (MF) category, the terms with the most unigenes were ion binding (336; 23.48%), molecular function (144; 10.06%), oxidoreductase activity (143; 9.99%), kinase activity (117; 8.18%), DNA binding (105; 7.34%), and transmembrane transporter activity (74; 5.17%).

#### 3.5.4. KEGG Pathways Mapping

**Figure 8:**
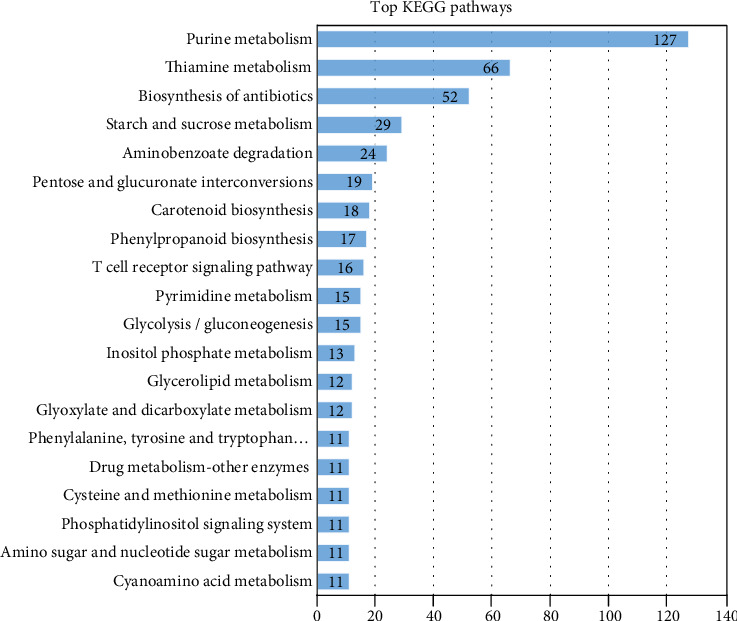
The top 20 KEGG pathways assignments in heat stress-responsive downregulated unigenes. The number of unigenes predicted to belong to each category is shown.

The KEGG pathways-based analysis indicated that 310 (21.13%) unigenes of the 1467 downregulated unigenes under heat stress obtained hits in the KEGG database, and those unigenes were associated with 183 enzymes and 100 KEGG pathways (Supporting Table 6). Of the 100 pathways, the top pathways are presented in Figure 8. Unigenes associated with Purine metabolism (127 unigenes) were most representative, followed by thiamine metabolism (66 unigenes) and biosynthesis of antibiotics (52 unigenes).

**Figure 9:**
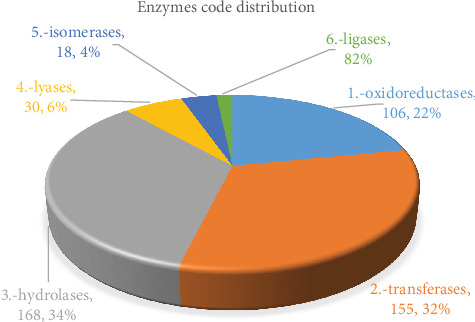
Enzyme code distribution of heat stress-responsive downregulated.

The 183 enzymes were further categorized into 6 main classes. As illustrated in Figure 9, Hydrolases enzymes represented the largest number of unigenes (168; 34.64%), followed by Transferases (155; 31.96%), Oxidoreductases (106; 21.86%), Lyases (30; 6.19%), Isomerases (18; 3.71%), and Ligases which represented the lowest number of unigenes (8; 1.65%). These 6 classes were categorized again to subclasses.

#### 3.5.5. Downregulated TFs

**Figure 10:**
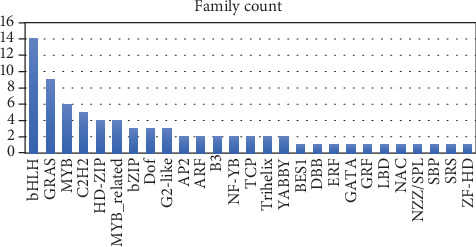
Distribution of downregulated transcription factors (TFs) under heat.

A total of 76 downregulated TFs were identified from DEGs. These downregulated TFs belong to 27 TF families. The bHLH family represented the most of TFS (14), followed by GRAS (9), MYB (6), C2H2 (5), HD-ZIP (4), and MYB_related (4), then the other families included 3 TFS or less (Figure 10). The description for the best hit of these TFs against *Arabidopsis thaliana* are presented in Supporting Table 7.

## 4. Discussion

Heat stress influences plant growth and development and can reduce crop yield [19]. To alleviate the effects of heat stress, it is critical to contrive plants that can withstand environmental challenges.

RNA-Seq is a sturdy technology that has been used to get genome-wide estimates of the relative expression of genes, as well as to identify genes, hormones, and processes which are participated in the response of leguminous plants to heat stress such as *Glycine max* and *Cicer arietinum* [25, 26]. Guar becomes an important forage crop and used industrially. Despite the few studies that have been published on heat stress in guar at physiological and morphological levels, its underlying molecular mechanism remains obscure. In this study, we examined the genes that were responsive to high temperatures (42°C) at preflowering stage for the leaves of heat-tolerant guar accession “PWP 5595” using RNA-seq compared with the control (25°C).

The differential expression analysis of RNA-seq data revealed that cutting the hierarchically clustered gene tree at 60% height of the tree resulted into partitioning genes into two clusters which represent 1551 upregulated and 1466 downregulated DEGs. Our results are in the range of previous studies of heat stress-responsive DEGs. For instance, the range of DEGs varied from 607 [11] to 11471 [22].

Gene ontology (GO) is a major bioinformatics initiative to unify the representation of gene and gene product attributes across all species [42]. Its enrichment analysis allowed us to effectively identify key biological processes that were associated with heat stress response. Out of these DEGs identified in our current study, 1030 (66.41%) upregulated and 1135 (77.37%) downregulated unigenes were assigned a GO classification. These findings are higher than Li et al. [19], who found that only 27% of their candidate genes were assigned a GO classification.

Protein sequence analysis & classification (InterProScan; IPS) is a tool that allows sequences (protein and nucleic) to be scanned against InterPro's signatures. Our collection of heat-responsive DEGs were blasted to the 14 databases of InterPro consortium. In upregulated DEGs collection, we found 301 protein families, 551 domains, 15 repeats, and 3 sites. On the other side, our downregulated DEGs matched with 353 families, 647 domains, 109 repeats, and 5 sites. Cytochrome P450 enzymes are a superfamily of haem-containing monooxygenases that are found in all kingdoms of life [43]. In plants, cytochrome P450s are important for the biosynthesis of several compounds such as plant hormones, defensive compounds, secondary metabolites, lignin, and fatty acids [44]. Annotations of the plant genome suggest that cytochrome P450 genes account for as much as 1% of plant genes. The number and variability of P450 genes are partly responsible for the host of bioactive compounds [45]. Cytochrome P450 119 (CYP119) that isolated from *Sulfolobus acidocaldarius* are thermophilic enzymes evolved to function at high temperatures [46]. In our study, Cytochrome P450 and Cytochrome P450, E-class, group I matched with the highest number of upregulated DEGs (18 and 16 unigenes, respectively). Oppositely, 12 and 11 downregulated DEGs were coded to Cytochrome P450 and Cytochrome P450, E-class, and group I families, respectively. This contrasting in gene expression is either due to the translation of different proteins of different roles within these two families or due to a disturbance caused by heat stress. Aspartic peptidase family A1, also known as the pepsin family, contains peptidases with bilobed structures [47]. In plants, phytepsin (EC: 3.4.23.40) Seed storage proteins and nepenthesin degradation (EC 3.4.23.12) from a pitcher plant digest insect protein. Family A5 contains thermopsin, an endopeptidase found in thermophilic archaea only [48]. Nine of our upregulated DEGs under heat stress were in match with this family, suggesting a high expression of thermophilic enzymes. Members of major facilitator, sugar transporter-like family include sugar transporters, which are responsible for the binding and transport of various carbohydrates, organic alcohols, and acids in a vast number of prokaryotic and eukaryotic organisms [49]. Most but not all members of this family catalyze sugar transport [50]. Seven of our upregulated DEGs matched with this family pointing out the necessity of plant to high transportation of sugar under heat stress conditions.

Heat shock proteins (HSPs) are a group of heat shock-induced proteins found in virtually every living organism, from bacteria to humans [51]. In plants, the *HSP* gene family members play important roles in developmental processes, as well as different kinds of environmental stress conditions, such as heat stress [52]. They maintain proper protein folding within the cell and assist in the folding of nascent polypeptide chains and are also involved in the refolding of denatured proteins following proteotoxic stress [53]. HSPs are classified into five major families: Hsp100, Hsp90, Hsp70, Hsp60, and the small Hsps [54]. Small heat shock protein HSP20 is a protein family having an average molecular weight of 20 Kd [55]. Hsp20 proteins seem to form large hetero-oligomeric aggregates. In our study, 16 DEGs related to this family were upregulated in response to heat stress. Similarly, Wan et al. [22] reported that 15 genes represented various small HSPs. Hsp90 chaperones are unique in their ability to regulate a specific subset of cellular signalling proteins that have been implicated in disease processes, including intracellular protein kinases, steroid hormone receptors, and growth factor receptors [56]. Seven unigenes of our upregulated DEGs were coded for HSPs, which is in alignment with Wan et al. [22], who found 3 genes coded for HSP90. Heat shock proteins 70 (HSP70) chaperones help to fold many proteins. Hsp70 assisted folding involves repeated cycles of substrate binding and release. Hsp70 activity is ATP dependent [57]. They have an average molecular weight of 70 kDa (Craig, 1989). There are many proteins in most species that belong to the Hsp70 family, and some are expressed only under stress conditions (strictly inducible) [58]. Six unigenes in our collection of upregulated DEGs were coded for HSP70. Our findings are similar to Wan et al.'s [22] results, who found 18 genes coded for HSP70 in Carnation (*Dianthus caryophyllus* L.) under heat stress. Heat shock factors (HSFs) induce transcription of heat shock genes following stress and in response to developmental signals [59]. They recognize cis-acting promoter elements composed of variations of an inverted repeat called heat shock elements (HSE) [60]. HSF is present in a dormant state under normal circumstances; it is stimulated by the initiation of trimerisation and high-affinity DNA binding and by disclosure to transcriptional domains [53]. Out of our upregulated DEGs, 11 unigenes were coded for HSFs.

Upregulated DEGs were also coded under heat stress to a considerable number of heat shock protein (HSP) domains including (IPR020575) Heat shock protein Hsp90, N-terminal (7 unigenes), (IPR029047) Heat shock protein 70kD, peptide-binding domain (6 unigenes), (IPR000232) Heat shock factor (HSF)-type, DNA-binding (6 unigenes), (IPR029048) Heat shock protein 70kD, C-terminal domain (5 unigenes), (IPR001305) Heat shock protein DnaJ, cysteine-rich domain (2 unigenes), and (IPR006636) Heat shock chaperonin-binding (1 unigene). Two upregulated DEGs of our collection were coded to the activator of Hsp90 ATPase homologue family. This family includes eukaryotic, prokaryotic, and archaeal proteins which have similarity to a 90 kDa heat shock protein ATPase homologue 1 C-terminal region of human activator (AHSA1/p38, O95433). This protein is reported to interfere with Hsp90's middle domain and enhance its activity in ATPase [61]. It is possibly a general upregulator of Hsp90 activity, which contributes in particular to its efficiency under conditions of increased stress [62]. Protein kinases alter other proteins by putting phosphate groups to them. Ethylene is an endogenous plant hormone that influences many aspects of plant growth and development [63]. In our upregulated DEGs collection, 46 and 43 unigenes were coded for protein kinase-like domain and Protein kinase domain, respectively, indicating an increased requirement to signaling and regulatory processes under heat stress conditions.

Ethylene is an endogenous plant hormone that influences many aspects of plant growth and development. Some defense-related genes triggered by ethylene include a cis-regulatory element identified as the Ethylene-Responsive Element (ERE) [64]. Twenty-nine of our upregulated DEGs were coded to the AP2/ERF domain. *Arabidopsis thaliana* and *Zea maize* abscisic acid- (ABA-) insensitive 4 (ABI4) proteins contain an AP2/ERF domain. They bind to an element similar to GCC found in ABA-responsive genes [65].

UDP-glucuronosyl/UDP-glucosyltransferase family currently consists of plants Flavonol O (3)-glucosyltransferase (EC: 2.4.1.91), an enzyme that catalyzes the transfer of glucose from UDP-glucose to a Flavonol. This reaction is essential and one of the last steps in anthocyanin pigment biosynthesis [66, 67]. Our results revealed that 11 and 17 DEGs, which matched with this family, were up- and downregulated, respectively, indicating an imperfection in the anthocyanin pigment biosynthesis under heat stress.

The light-harvesting complex (LHC) consists of chlorophylls A and B and the chlorophyll A-B binding protein [68]. The N terminus of the chlorophyll A-B binding protein reaches into the stroma where it involves the adhesion of granular membranes and photo-regulates the threonin residues by reversible phosphorylation [69]. Chlorophyll A-B binding protein family also involves the photosystem II protein PsbS, which has a role in energy-dependent quenching that rises thermal dissipation of excess absorbed light energy in the photosystem [70]. Ten downregulated DEGs matched with Chlorophyll A-B binding protein family. Furthermore, at least one downregulated gene matched to proteins related to the photosynthesis process. For instance, (IPR000549) Photosystem I PsaG/PsaK protein (2 unigenes), (IPR005610) Photosystem II Psb28, class 1 (1 unigene), (IPR017493) Photosystem I reaction centre subunit psaK, chloroplastic (1 unigene), (IPR017494) Photosystem I PsaG, plant (1 unigene), (IPR008148) DNA photolyase class 2 (1 unigene), and (IPR009518) Photosystem II PsbX (1 unigene). These findings are similar to the results of Song [71] et al. (2014) and pointing out an adverse effect of heat stress on the photosynthesis process. Expansions are unusual proteins that mediate cell wall extension in plants [72]. They are thought to act as a kind of chemical grease, enabling polymers to slide past each other by disrupting noncovalent hydrogen bonds, which hold many wall polymers together. This process is not deteriorating and therefore does not weaken the wall, that otherwise could collapse during growth under internal pressure. Seven downregulated DEGs matched with the expansin/Lol pI family. Protein kinases modify other proteins by chemically adding phosphate groups to them. This process is fundamental to most signaling and regulatory processes in the eukaryotic cell [63]. In our collection, 83 and 79 downregulated DEGs were matched with protein kinase-like domain and protein kinase domain, respectively. Proteins containing Leucine-rich repeats (LRR) are involved in a range of biological processes, including DNA repair, disease resistance, cell adhesion, recombination, RNA processing, signal transduction, apoptosis, transcription, and the immune response [73]. Sixty-one downregulated DEGs matched with the Leucine-rich repeat domain.

Transcription factors (TFs) (or sequence-specific DNA-binding factor) are proteins that control the rate of transcription of genetic information from DNA to messenger RNA, by binding to a specific DNA sequence [74]. The function of TFs is to regulate (turn on and off) genes in order to make sure that they are expressed in the right cell at the right time and in the right amount throughout the life of the cell and the organism. TFs work alone or with other proteins in a complex, by promoting (as an activator) or blocking (as a repressor) the recruitment of RNA polymerase to specific genes [75]. In our study, 87 and 76 up- and downregulated DEGs were coded to transcription factors, respectively. Heat shock factors (HSFs) are the transcription factors that regulate the plant heat stress response [76]. They play crucial roles in thermotolerance by binding the promoter region of HSP genes to the cis-acting regulatory elements called heat shock elements (HSEs) [77]. Plant HSFs are divided into three classes, HSFAs, HSFBs, and HSFCs [78]. Out of our collection under heat stress, 6 upregulated DEGs were coded to HSFs including heat shock factor 4, heat shock transcription factor A4A, heat shock transcription factor A6B, heat shock transcription factor B2A, and heat shock transcription factor B3. HSF4 encodes a protein whose sequence is similar to heat shock factors that regulate the expression of heat shock proteins, and its transcript level is increased in response to heat shock. HSFA4A encodes a member of the HSFs family that is a substrate of the MPK3/MPK6 signaling and regulates stress responses. HSFA6B has been reported to play a pivotal role in ABA response and thermotolerance [12], which is in alignment with our findings. NAC transcription factors are one of the biggest families of transcriptional regulators in plants, and it was suggested that members of the NAC gene family play a significant role in regulating transcriptional reprogramming associated with plant stress responses. The role of HSFB2A in heat stress tolerance has been approved in many studies. For instance, Ashraf [79] found that HSFB2A positively regulates the response to high temperature in *Capsicum annuum*. Furthermore, Kanchiswamy et al. [80] reported that HSFB2A is involved in the heat response signaling pathway in Arabidopsis. Moreover, Wunderlich et al. [81] found that HSFB2A involved in the gametophyte development of Arabidopsis thaliana, and its expression is controlled by a heat-inducible long noncoding antisense RNA. APETALA2/Ethylene-responsive factor (AP2/ERF) is one of the most eminent families of genes in plants, which play an important role in regulating plant growth and responses to various stresses [82]. After finding the tobacco ERFs, many proteins in the ERF family were identified and implicated in many diverse functions in cellular processes, such as hormonal signal transduction, response to biotic and abiotic stresses, regulation of metabolism, and in developmental processes in various plant species [83]. In our study, we recorded 21 upregulated DEGs coded for the ERF family. The best hit of these genes against *A. thaliana* showed that 11 genes were coded to ERF family protein, 6 genes to ethylene response factor 1 and ethylene-responsive element binding factor 1, 4 genes related to AP2, and one gene to cytokinin response factor 4. WRKY transcription factors are key regulators of many processes in plants including the responses to biotic and abiotic stresses, senescence, seed dormancy and seed germination, and some developmental processes [84]. Nine of our upregulated DEGs were coded to WRKY TFS including WRKY DNA-binding protein (WRKY23, WRKY33, WRKY51, WRKY60, and WRKY69) and WRKY family protein. WRKY DNA-binding protein 33 (WRKY33) found to be functioning with WRKY25 and WRKY26 as a positive regulator of plant thermotolerance by partially participating in the ethylene-response signal transduction pathway [85]. NAC transcription factors are one of the largest families of transcriptional regulators in plants, and members of the *NAC* gene family have been suggested to play important roles in the regulation of the transcriptional reprogramming associated with plant stress responses [86]. There is increasing evidence that NAC proteins are involved in responses to heat stress. For example, the NAC TF gene (*ONAC063*) in rice roots responds to a combination of high-temperature stress [87]. Additionally, a gene expressing a CsNAM-like protein is induced by heat in tea plants (*Camellia sinensis*) [88]. Moreover, the transgenic Arabidopsis plants overexpressing *ANAC042* show increased tolerance to heat stress when compared to the wild-type plants [89]. In our study, 7 upregulated DEGs were coded to the NAC family. The best hit of these genes against *A. thaliana* showed that these genes were coded to the NAC domain-containing protein (17, 36, and 102), NAC family protein, NAC transcription factor-like 9, and NAC-like, activated by AP3/PI. MYB TF family of proteins is large and involved in controlling various processes like responses to biotic and abiotic stresses, development, differentiation, metabolism, defense, etc. [90]. El-Kereamy et al. [91] concluded that the overexpression of OsMYB55 improved rice (*Oryza sativa*) plant tolerance to high temperature. In our collection of upregulated DEGs, there are 6 unigenes that encoded to MYB family.

## 5. Conclusions

In the current study, we investigated the genes that differentially expressed responsive to heat stress and their metabolic pathways. Our results uncovered 1551 up- and 1466 downregulated differentially expressed genes responsive to heat stress. Of those, 200 up- and 72 downregulated genes could be considered as new genes exclusive to guar responsive to heat stress. Cytochrome P450, small heat shock protein HSP20, heat shock transcription factor family, heat shock protein Hsp90 family, and heat shock protein 70 family were the most upregulated protein families. Heat shock factor 4, heat shock transcription factor A6B, heat shock transcription factor B3, heat shock transcription factor A4A, heat shock transcription factor B2A, and heat shock factor 6 were upregulated responsive to heat stress. Resulting data will be helpful to understand the molecular behaviour of plants induced by heat stress. The new putative and membranes' genes might be useful for future researches.

## Figures and Tables

**Table 1:** Raw data statistics and quality assessment.

Treatment/replicate	Total read bases (bp)	Total reads (pairs)	GC (%)	Q30 (%)
C1	3,682,737,548	18,231,374	44.77	95.08
C2	3,746,599,646	18,547,523	44.55	94.58
C3	3,881,717,446	19,216,423	44.49	94.98
H1	3,589,147,716	17,768,058	44.2	94.97
H2	3,541,905,774	17,534,187	44.11	94.93
H3	3,922,224,910	19,416,955	43.98	95.3

GC (%): GC content; Q30 (%): ratio of reads having a Phred quality score of over 30; C: control; H: heat treatment.

**Table 2:** Examples of upregulated heat-responsive genes in guar.

Putative gene name	Description	Length (bp)
TRINITY_DN35257_c0_g1	Heat shock 70 kDa	2645
TRINITY_DN13155_c0_g1	Heat-stress-associated 32	2460
TRINITY_DN25460_c0_g1	kDa class II heat shock-like	908
TRINITY_DN19883_c1_g15	Heat stress transcription factor A-4c-like	1740
TRINITY_DN8056_c0_g1	kDa class III heat shock	1364
TRINITY_DN5986_c0_g1	kDa class I heat shock-like	1098
TRINITY_DN18122_c0_g2	kDa class I heat shock-like	896
TRINITY_DN18254_c0_g4	kDa class IV heat shock	1178
TRINITY_DN4388_c0_g1	Small heat shock chloroplastic-like	1126
TRINITY_DN14463_c0_g1	Heat shock cognate 80	2759
TRINITY_DN15735_c0_g2	Small heat shock chloroplastic	2292
TRINITY_DN11506_c0_g1	Heat shock cognate 70 kDa 2	2942
TRINITY_DN3531_c0_g1	kDa class IV heat shock	1024
TRINITY_DN20694_c0_g1	Heat shock 83	2644
TRINITY_DN2570_c0_g1	Class I heat shock-like	812
TRINITY_DN16702_c1_g1	Heat shock factor HSF30	2237
TRINITY_DN15088_c0_g1	kDa class I heat shock	2499
TRINITY_DN19733_c0_g1	Heat shock 83	2536
TRINITY_DN25024_c0_g1	Heat stress transcription factor B-2a-like	423
TRINITY_DN12431_c0_g1	Small heat shock	1291
TRINITY_DN15088_c1_g1	kDa class I heat shock	562
TRINITY_DN12794_c0_g2	Heat stress transcription factor B-2b	2433
TRINITY_DN38964_c0_g1	kDa class II heat shock	895
TRINITY_DN18629_c0_g2	Heat shock HSP 90-beta	2381
TRINITY_DN8034_c0_g1	Class I heat shock	849
TRINITY_DN19698_c0_g1	Heat shock 90- chloroplastic	3176
TRINITY_DN9349_c0_g1	kDa class II heat shock-like	940
TRINITY_DN10493_c0_g1	Heat shock cognate 71 kDa	2054
TRINITY_DN7499_c0_g1	kDa class I heat shock-like	834
TRINITY_DN26877_c0_g1	Heat stress transcription factor B-2a-like	743
TRINITY_DN24551_c0_g1	Activator of 90 kDa heat shock ATPase homolog	1771
TRINITY_DN8550_c0_g1	Heat shock factor HSF24-like	1680
TRINITY_DN11358_c0_g2	Heat stress transcription factor C-1	884
TRINITY_DN10430_c0_g1	Heat shock 90- mitochondrial	2982
TRINITY_DN12677_c0_g1	kDa heat shock peroxisomal	1302
TRINITY_DN35298_c0_g1	kDa class I heat shock-like	1156
TRINITY_DN16768_c1_g1	Heat stress transcription factor A-6b-like	1616
TRINITY_DN40620_c0_g1	Heat shock 70 kDa	2605
TRINITY_DN18528_c0_g5	Heat stress transcription factor B-3	1280
TRINITY_DN19073_c1_g2	Heat stress transcription factor B-2a-like	1936
TRINITY_DN18254_c0_g3	Activator of 90 kDa heat shock ATPase homolog	2328
TRINITY_DN16913_c0_g5	DNAJ heat shock N-terminal domain-containing	749
TRINITY_DN37813_c0_g1	kDa class II heat shock-like	239
TRINITY_DN14498_c0_g1	Heat shock factor HSF24	2074
TRINITY_DN20386_c0_g11	kDa heat shock mitochondrial	1530
TRINITY_DN19503_c0_g3	Heat shock 70 kDa mitochondrial	3512
TRINITY_DN7426_c0_g1	Heat stress transcription factor A-3-like	3147
TRINITY_DN19828_c0_g2	kDa class IV heat shock	1262
TRINITY_DN19733_c0_g4	Heat shock 83	3248
TRINITY_DN9818_c0_g1	Heat shock 70 kDa 8	3021

## Data Availability

The raw sequence data has been deposited at the NCBI Short Read Archive (SRA) with accession numbers (SRR10120601, SRR10120602, SRR10120603, SRR10120610, SRR10120611, and SRR10120612).

## Supplementary Materials

Supplementary Materials Supporting Figure 1: pairwise comparisons of replicate log (CPM) values in the control (GC). Supporting Figure 2: pairwise MA plots in control (GC). Supporting Figure 3: the sum of mapped genes in the control (GC). Supporting Figure 4: a replicate Pearson correlation heatmap in the control (GC). Supporting Figure 5: pairwise comparisons of replicate log (CPM) values in the heat stress (GH). Supporting Figure 6: pairwise MA plots in heat stress (HC). Supporting Figure 7: the sum of mapped genes in the heat stress (GH). Supporting Figure 8: a replicate Pearson correlation heatmap in heat stress (GH). Supporting Figure 9: sample correlation matrix between the control (GC) and heat stress (GH). Supporting Figure 10: principal component analysis of the control (GC) and heat stress (GH). Supporting Figure 11: distribution of genes according to fold-change (FC), counts, and FDR. Supporting Figure 12: the clustered heatmap showing the correlation matrix of DEGs between the heat stress and the control at *p* value of 0.001 and FC of 2. Supporting Figure 13: the clustered heatmap of DEGs of heat stress (GH) via control (GC) at *p* value of 0.001 and FC of 2. Supporting Figure 14: the expression patterns for the genes in each cluster under the heat stress and the control. Supporting Figure 14: upregulated genes in starch and sucrose metabolism pathway under heat stress. Supporting Table 1: Pearson correlation values between the replicates of control (GC) and heat stress (GH). Supporting Table 2: principal component analysis values for the replicates of control (GC) and heat stress (GH). Supporting Table 3: Pearson correlation matrix values between the replicates of control (GC) and heat stress (GH) based on the set of DE genes. Supporting Table 4: list of KEGG pathways assignments in upregulated unigenes responsive genes under heat stress. Supporting Table 5: best hit of upregulated transcription factors (TFs) in Arabidopsis thaliana. Supporting Table 6: list of KEGG pathway assignments in downregulated unigenes responsive genes under heat stress. Supporting Table 7: best hit of upregulated transcription factors (TFs) in Arabidopsis thaliana.
